# Acute ST-segment elevation myocardial infarction due to extrinsic compression of left coronary artery from pulmonary epithelioid hemangioendothelioma: A case report

**DOI:** 10.1097/MD.0000000000018158

**Published:** 2019-11-27

**Authors:** Xiaojia Luo, Ningying Song, Sen He, Xin Wei, Yuan Feng, Yong He, Xiaoping Chen

**Affiliations:** a The Second People's Hospital of Chengdu, Chengdu 610017; b Department of Cardiovascular Medicine; c Department of Otorhinolaryngology-Head and Neck Surgery, West China Hospital, Sichuan University, Chengdu 610041, China.

**Keywords:** acute myocardial infarction, extrinsic compression, pulmonary epithelioid hemangioendothelioma, ST-segment elevation myocardial infarction

## Abstract

**Rationale:**

Acute myocardial infarction is usually caused by coronary atherosclerotic plaque disruption (rupture or erosion), also including other uncommon etiologies. Pulmonary epithelioid hemangioendothelioma (PEH) is a rare low to intermediate malignant vascular tumor originating from vascular endothelial cells. Here, we report a rare case of acute ST-segment elevation myocardial infarction (STEMI) due to extrinsic compression of left coronary artery from PEH.

**Patient concerns:**

A 63-year-old woman with pulmonary nodules received left pulmonary nodulectomy, and the pathological examination indicated PEH. Five months after the pulmonary nodulectomy, the patient was admitted due to progressive dyspnea.

**Diagnosis:**

Electrocardiography showed the obvious ST-segment elevation in the leads I, aVL, and V1–3, and laboratory tests revealed the elevated level of cardiac troponin T. Emergent coronary angiography and the contrast-enhanced computed tomography scan conformed STEMI due to extrinsic compression of left coronary artery from PEH.

**Interventions:**

The patient did not undergo further therapy after the pulmonary nodulectomy. During the present hospitalization, she received basic life support and nutritional support treatment.

**Outcomes:**

The patient deteriorated rapidly into multi-organ failure and eventually died.

**Lessons:**

Acute STEMI could be caused by extrinsic compression of the coronary artery from the mass effects of PEH, and active therapy and close follow-up should be considered for patients with PEH.

## 1 Introduction

Acute myocardial infarction (AMI) is defined pathologically as myocardial cell death due to prolonged ischemia. The first ultrastructural injuries become visible as early as 10–15 minutes after the onset of ischemia.^[1]^ In clinical practice, identifying the specific causes of ischemia could help us perform appropriate reperfusion therapy in a timely and accurate manner, in order to reduce ischemic injury to the myocardium.^[2–4]^ AMI is usually caused by coronary atherosclerotic plaque disruption (rupture or erosion), also including other uncommon etiologies.^[2]^ As one of the uncommon causes, cancer can result in AMI by different mechanisms, including cancer, chemotherapy, and radiation therapy effects.^[5]^ Tumor compression or invasion of a major coronary artery leading to occlusion has also been reported, but is rare.^[5,6]^ Pulmonary epithelioid hemangioendothelioma (PEH) is a rare low to intermediate malignant vascular tumor, which commonly affects noncardiac organs in the previous literatures.^[7]^ Here, we firstly report a rare case of acute ST-segment elevation myocardial infarction (STEMI), in which coronary obstruction was not due to plaque rupture but due to compression of the coronary artery from PEH.

## 2 Case report

**Figure 1:**
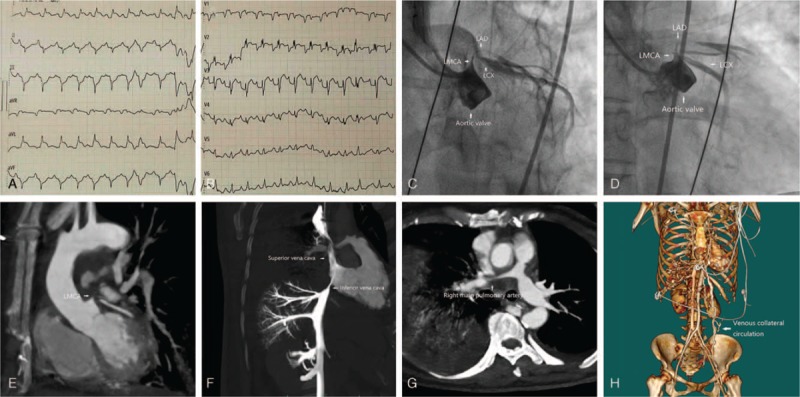
Electrocardiogram, coronary angiography, and contrast-enhanced CT. (A and B) ECG on admission showed ST segment elevation in leads I, aVL, and V1–V3. (C and D) Emergent coronary angiography demonstrated severe stenosis of coronary artery and the abnormal changes in the aortic valves. (E–G) Contrast-enhanced CT scan showed that the LMCA, LAD, and LCX, as well as the proximal superior/inferior vena cava and the right main pulmonary artery, were compressed by the mediastinal soft tissues, resulting in severe stenosis of these vessels. (H) Venous collateral circulation dilatation due to severe stenosis of the inferior vena cava was observed. CT = computed tomography, ECG = electrocardiography, LAD = left anterior descending coronary artery, LCX = left circumflex coronary artery, LMCA = left main coronary artery.

In September 2018, a 63-year-old woman was admitted to our hospital due to progressive dyspnea over the past month. Vital signs: weakness, blood pressure 88/50 mm Hg, respiratory rate 28 bpm, heart rate 146 bpm, and temperature 36.5°C, as well as oxygen saturation 88%. Electrocardiography (ECG) showed the obvious ST-segment elevation in the leads I, aVL, and V1–3 (Fig. 1A and B). Laboratory tests revealed the elevated level of cardiac troponin T (576.7 ng/L; reference range: 0–14 ng/L). Emergent coronary angiography demonstrated 95% distal occlusion of the left main coronary artery (LMCA), 95% ostium occlusion of the left anterior descending coronary artery (LAD) and left circumflex coronary artery (LCX), and the abnormal changes in the aortic valves (Fig. 1C and D). We then performed the contrast-enhanced computed tomography (CT) scan promptly, which showed the compression of mediastinal soft tissues on the LMCA, LAD, and LCX, as well as the proximal superior/inferior vena cava and the right main pulmonary artery, resulting in severe stenosis of these arteries and veins (Fig. 1E–G). Venous collateral circulation dilatation due to severe stenosis of the inferior vena cava was observed (Fig. 1H). Further inquiry revealed that the patient complained of a cough 5 months ago in the local hospital, and CT scan showed pulmonary nodules; she received left pulmonary nodulectomy. The pathological examination indicated PEH (immunohistochemical staining: ERG+, CD31+, CD34±, CAMTA1+, TFE3−, EMA+/−, CK−, TTF1−, P63−, Ki67+). She did not undergo further therapy after the pulmonary nodulectomy. Unfortunately, the patient deteriorated rapidly into multi-organ failure and eventually died.

## 3 Discussion

AMI is attributable to an occluded coronary artery in almost 90% of patients. However, not all cases of AMI are because of coronary artery occlusion. AMI that occurs in the absence of obstructive coronary artery disease on angiography has been termed myocardial infarction with nonobstructive coronary arteries (MINOCA).^[8]^ Potential causes include coronary artery spasm, emboli, radiation associated fibrosis, coronary artery vasculitis, myocardial oxygen demand-supply disproportion, external compression, and noncardiac pathology.^[8,9]^ Among these potential causes, external compression of neoplasms can lead to pain, ECG changes, and a rise in troponin, and most neoplasms that are responsible for myocardial infarction relate to metastatic deposits to the heart (e.g., lung, breast, and thymoma).^[9]^ Although external compression could be successfully treated with percutaneous coronary intervention,^[10]^ the prognosis is very poor.^[5]^

PEH is a rare low to intermediate malignant vascular tumor and represents 12% of all epithelioid hemangioendothelioma cases. Single-organ involvement is seen in 19% of cases.^[11,12]^ There is still no characteristic clinical or biological marker for PEH, which typically occurs among young patients and is more common in women than in men. In most cases, the disease is an incidental finding observed during physical examinations of asymptomatic patients but it also can be accompanied by symptoms such as coughing, shortness of breath, or hemoptysis.^[7,13]^ The prognosis can vary considerably. Slow progression or growth over long periods and spontaneous regression might occur, especially in asymptomatic patients.^[7,13,14]^ In patients with clinical symptoms such as weight loss, anemia, pulmonary symptoms, or pleural hemorrhagic effusions, the disease could show rapid progression and be accompanied by various patterns of tumor growth.^[7]^ The median survival time was usually less than 1 year.^[15]^ There is no established standard treatment for PEH. Observation might be an option for patients without symptoms.^[13]^ For symptomatic patients, or those who experience disease exacerbation, specific therapies are recommended. Radiotherapy has proven to be ineffective for PEH, and chemotherapy with immunostimulants is recommended for patients with disseminated disease. Lung transplantation can be an option. When tumor resection is feasible, surgery is usually considered the treatment of choice. However, complete surgical resection is usually not feasible.^[7,16]^

In this case, the patient was diagnosed with PEH by the imaging and immunohistochemical staining 5 months ago. She only received the pulmonary nodulectomy. PEH progressed rapidly and resulted in progressive dyspnea. She finally presented as acute STEMI and died with multi-organ failure. Our case suggested that acute STEMI could be caused by extrinsic compression of the coronary artery from the mass effects of PEH. Active therapy and close follow-up should be considered for patients with PEH.

## Author contributions

**Data curation:** Yuan Feng.

**Funding acquisition:** Xiaoping Chen.

**Resources:** Xin Wei.

**Supervision:** Yong He, Xiaoping Chen.

**Validation:** Yong He, Xiaoping Chen.

**Writing – original draft:** Sen He.

**Writing – review & editing:** Xiaojia Luo, Ningying Song.
